# Prevalence of psychological symptoms in low and high-risk pregnant women: A cross-sectional study

**DOI:** 10.18502/ijrm.v23i4.18781

**Published:** 2025-06-10

**Authors:** Maryam Dalili, Ali Mehdizadeh, Maryam Nodinnejad, Fatemeh Karami Robati

**Affiliations:** ^1^Department of Obstetrics and Gynecology, School of Medicine, Kerman University of Medical Sciences, Kerman, Iran.; ^2^Maternal-Fetal Medicine Research Center, Shiraz University of Medical Sciences, Shiraz, Iran.; ^3^Kerman University of Medical Sciences, Kerman, Iran.; ^4^Clinical Research Development Unit, Afzalipour Hospital, Kerman University of Medical Sciences, Kerman, Iran.

**Keywords:** Psychiatric disorders, Pregnant women, High-risk pregnancy.

## Abstract

**Background:**

Common psychological disorders during pregnancy can have obvious harmful effects on both mother and fetus.

**Objective:**

This study aimed to investigate the prevalence of psychological symptoms in low and high-risk pregnant women.

**Materials and Methods:**

This cross-sectional study was conducted on 400 low-risk and high-risk pregnant women in Afzalipour hospital, Kerman, Iran from December 2017–2018. Participants were selected by census method, and the data collection tool was a 90-item questionnaire named Symptom Checklist-90.

**Results:**

The mean age of pregnant women was 29.1 ± 6.8 yr. 7.6% had gestational diabetes mellitus, 5.9% had pregnancy hypertension, 6.6% had a history of in vitro fertilization, and 17.5% had a history of one miscarriage. 61.2 and 65.5% of high-risk women had depression and anxiety, respectively. A significant difference was observed between low-risk and high-risk women in terms of depression (p = 0.019), anxiety (p = 0.049), and aggression (p = 0.013), and the frequency of these variables was higher in high-risk women than in low-risk women.

**Conclusion:**

According to age, education, and gestational period, the differences between 2 groups (low-risk and high-risk) were significant. Compared with low-risk women, high-risk pregnant women reported a higher prevalence of psychological symptoms in 10 factors. High-risk pregnant women had a significantly higher prevalence of somatization symptoms, obsessive-compulsive symptoms, depression symptoms, anxiety symptoms, hostility symptoms, and paranoid ideation than low-risk women. Therefore, educational programs during pregnancy for high-risk women can be useful.

## 1. Introduction

Psychological disorders during pregnancy have extensive negative effects on mothers and infants, such as increased risk of premature birth, low birth weight, postpartum depression, self-harm or suicide, not seeing the doctor, difficulty in performing common activities, inappropriate diet, and smoking and alcohol consumption. These disorders during pregnancy are considered as significant predictors of postpartum depression (1). There is evidence that psychological disorders, especially anxiety and depression in mothers and the negative impact they have on child development, are serious public health concern (2).

Childhood is a significant stage of child development that is directly affected by the infant's comfort and it depends on the quality and quantity of care that the primary caregiver, usually the mother, receives. Depressed mothers have less contact with their infants, which leads to unsure attachment in children. Some studies have indicated that children born to mothers with mental disorders have weaker cognitive-motor and emotional-social development than children born to mothers with good mental health (3). Other researchers have associated major depressive disorder and anxiety during pregnancy with premature delivery, low birth weight, surgical delivery, and neonatal intensive care (4, 5).

The prevalence of depression during pregnancy ranges from 5.2–17.8% worldwide (6–11). The prevalence of anxiety disorder ranges from 0–10.5% (7, 9–14). Socio-cultural and methodological differences may cause some differences. Asians may under-report symptoms due to shame, and some risk factors for depression and psychological disorders may be relatively uncommon in Asia (15). For example, postpartum depression was more common among Indian and Chinese women who gave birth to girls than among women who gave birth to boys (16).

A study in Rio de Janeiro showed that one in 5 women attending antenatal care outpatient clinics of public hospitals had a case of International Classification of Diseases 10 ${}^{\text{th}}$ revision criteria for major depressive disorder in the past 12 months (17). In one study, the prevalence of psychological disorders in pregnant women was 20.2%. Improved knowledge about mental disorders during pregnancy and the recognition of the most vulnerable groups may contribute to evidence-based public policies as well as to the development and provision of sufficient care for those in need (18).

Therefore, this study aimed to investigate the prevalence of psychological symptoms in low-risk and high-risk pregnant women.

## 2. Materials and Methods

### Study design and participants

This cross-sectional study was conducted on 400 pregnant women selected by the census method referred to the Afzalipour hospital, Kerman, Iran from December 2017–2018. Participants medical history was extracted from their medical records. Pregnant women with diabetes, high blood pressure, a history of frequent abortions, a history of premature birth, and a history of multiple births were considered as high-risk (n = 200). Pregnant women who had no history of specific diseases, and whose blood pressure and weight were within normal ranges, were considered as low-risk (n = 200). Each woman completed the Symptom Checklist-90 (SCL-90) questionnaire. Women aged between 22 and 35 yr and second trimester gestational age were included in the study. Women with a history of mental illness or taking psychiatric drugs were excluded from the study.

### Data collection

Self-reported mental health symptoms were assessed using the SCL-90 questionnaire. The validity and reliability of the Chinese version of the SCL-90 had been measured and confirmed in previous studies. This questionnaire consists of 90 questions that assess 10 main symptom factors, including somatization, obsessive-compulsive behavior, interpersonal sensitivity, depression, anxiety, hostility, phobic anxiety, paranoid thoughts, psychosis, and additional items (such as appetite and sleep) in the past week. Each of the 10 symptom factors includes 6–13 items. The questions in this tool were rated on a 5-point Likert scale (from “1 = not at all" to “5 = extremely"). A higher score indicated a greater frequency and severity of psychological symptoms. The average score of each factor was used as an indicator to assess mental health status. A factor score ≥ 2 was considered to indicate the occurrence of mental health problems in that factor. In this study, this scale indicated good reliability (Cronbach's alpha = 0.99) (19).

### Sample size

Using the following formula, the sample size was 384, which increased to 400 for better results. 


$$n=\frac{z_{1-\frac{\alpha }{2}}^{2}p^{\left(1-p\right)}}{d^{2}}$$

P = 0.10, d = 0.03, confidence interval = 95%

The P or the prevalence of each psychological disorder was determined based on previous studies in Tehran. The value of d, which represents the accepted error in estimating the prevalence of each disorder in pregnant women in the community, was determined based on previous studies. Z is the index of normal distribution based on the table, whose value is equal to 1.96 in the case of 2 domains.

### Ethical Considerations

This study was approved by the Ethics Committee of Kerman University of Medical Sciences, Kerman, Iran (Code: IR.KMU.AH.REC.1396.1864). Before starting the study, written informed consent was obtained from each patient.

### Statistical Analysis

Data were analyzed by using descriptive statistics (frequency, percentage, mean, and standard deviation), analytical (Chi-square and *t* test), and Statistical Package for the Social Sciences, version 20, SPSS Inc., Chicago, Illinois, USA.

## 3. Results

Of the 400 participants (200 cases in high-risk pregnant group and 200 cases in low-risk women's group), the average age in high-risk pregnant women (29.8 yr) was lower than in low-risk group (30.8 yr). Generally, participants were highly educated (99% in high-risk group and 95.5% in low-risk group had a university degree or above). More than half of the participants in both groups were in the third trimester (37% in high-risk group and 77% in low-risk group). Most pregnant women had a natural pregnancy (96.59%) and singleton pregnancy (97.56%). According to age, education, and gestational period the differences between 2 groups were significant (Table I).

Compared with low-risk women, high-risk pregnant women indicated a higher prevalence of psychological symptoms across the SCL-90 factors. Chi-square tests reported that high-risk pregnant women had a significantly higher prevalence of somatization symptoms (18.5 vs. 5.5%), obsessive-compulsive symptoms (23% vs. 14%), depression symptoms (18% vs. 9.5%), anxiety symptoms (16.5 vs. 6.5%), hostility symptoms (24% vs. 10.5%), and paranoid ideation (12% vs. 5.5%) than low-risk women (Table II).

After considering the confounding variables (age, education, and gestation period), high-risk pregnant women suffered from somatization symptoms (OR = 4.52, p = 0.006), anxiety symptoms (OR = 3.54, p = 0.01), and hostility symptoms (OR = 2.7, p = 0.02) than healthy women (Table III).

**Table 1:** Sociodemographic between high-risk and low-risk pregnant women

**Variables **		**High-risk (n = 200)**	**Low-risk (n = 200)**	**P-value**
Age (yr)*		29.8 ± 2.88	30.8 ± 3.78	0.03
	≤ 30**	130 (65)	101 (50.5)	0.003
	> 30**	70 (35)	99 (49.5)	
Education**				
	**High school or less**	2 (1)	9 (4.5)	0.03
	**University degree or above**	198 (99)	191 (95.5)	
Gestational period (trimester)**				
	**First**	46 (23)	2 (1)	< 0.001
	**Second**	80 (40)	44 (22)	
	**Third**	74 (37)	154 (77)	
*Data presented as Mean ± SD, *t* test. **Data presented as n (%), Chi-square test				

**Table 2:** The prevalence of psychological symptoms between high-risk and low-risk pregnant women

**Variables **	**High-risk (n = 200)**	**Low-risk (n = 200)**	**P-value**
**Somatization ≥ 2**	37 (18.5)	11 (5.5)	< 0.001
**Obsessive-compulsive symptoms ≥ 2**	46 (23)	28 (14)	0.02
**Interpersonal sensitivity ≥ 2**	21 (10.5)	16 (8)	0.38
**Depression ≥ 2**	36 (18)	19 (9.5)	0.01
**Anxiety ≥ 2**	33 (16.5)	13 (6.5)	0.002
**Hostility ≥ 2**	48 (24)	21 (10.5)	< 0.001
**Phobic anxiety ≥ 2**	33 (16.5)	26 (13)	0.32
**Paranoid ideation ≥ 2**	24 (12%)	11 (5.5%)	0.02
**Psychosis ≥ 2**	18 (9%)	9 (4.5%)	0.07
**Additional items ≥ 2**	31 (15.5%)	19 (9.5%)	0.07
Data presented as n (%), Chi-square test			

**Table 3:** Comparison of psychological symptoms among high-risk and low-risk pregnant women

**Variables **	**Crude**		**Adjusted**	
	**OR**	**P-value**	**OR**	**P-value**
**Somatization ≥ 2**	3.63	0.008	4.52	0.006
**Obsessive-compulsive symptoms ≥ 2**	1.83	0.10	2.11	0.083
**Interpersonal sensitivity ≥ 2**	1.36	0.52	1.42	0.53
**Depression ≥ 2**	2.02	0.09	1.86	0.2
**Anxiety ≥ 2**	2.89	0.02	3.54	0.01
**Hostility ≥ 2**	2.66	0.01	2.7	0.02
**Phobic anxiety ≥ 2**	1.34	0.45	1.73	0.22
**Paranoid ideation ≥ 2**	2.25	0.11	2.71	0.08
**Psychosis ≥ 2**	2.06	0.19	2.51	0.14
**Additional items ≥ 2**	1.70	0.21	1.96	0.17
Logistic regression				

## 4. Discussion

This study was conducted to compare the frequency of psychological symptoms in low-risk and high-risk pregnant women. The results demonstrated that no significant difference was observed between low-risk and high-risk women in terms of suffering from somatization, obsessive-compulsive disorder, sensitivity in mutual relationships, morbid fear, paranoid thoughts, and psychosis. Meanwhile, there was a significant difference between low and high-risk women in terms of depression, anxiety, and aggression. The frequency of these variables was higher in high-risk women than in low-risk women. In one study, Greek high-risk pregnant women had significantly higher levels of anxiety and depression than low-risk pregnant women (20).

In our study, the most common psychological symptoms during pregnancy were anxiety, depression, somatization, obsessive-compulsive disorder, and sensitivity in mutual relationships. The results of another study indicated that pregnant women had fewer psychological symptoms (depression, anxiety, insomnia, and post-traumatic stress disorder) than non-pregnant women (21). Similar results have been reported in previous studies, for example, a study illustrated that pregnant women suffer from depression less than non-pregnant women (22). However, another research demonstrated that the levels of depression, anxiety, and stress were similar in pregnant and non-pregnant women (23). In addition, conflicting results have been reported, for example, a Nigerian study demonstrated that anxiety disorders are more common among pregnant women than non-pregnant women (39% vs. 16.3%) (24). These different results may be due to differences in the study population, instruments, and diagnostic criteria. Compared to pregnant women, non-pregnant women may have fewer opportunities to communicate with medical staff, and because of isolation they are more frustrated with their limited activities (21).

In a study, depression was the most common mental disorder reported (25), which was consistent with national and international studies (26, 27) related to public health. The emotional burden of depression on pregnant women can endanger their mental and physical health, be a risk factor for postpartum depression, and endanger the growth of the fetus (28). In addition, a literature review indicated that the presence of this disorder during pregnancy increases the risk of complications such as pre-eclampsia and premature birth (29).

The prevalence of depression during pregnancy has been reported to range from 13.5–42% (30–32). In our study, the prevalence of depression in high-risk women was equal to 70.7%, and in low-risk women, it was 62.6%. This difference can be due to the difference in cultural and social environment. In addition, the reason for the high prevalence of depression can be explained by the low average age of the participants in the above study compared to other studies.

Because in young mothers who have the most vulnerability during pregnancy, the need for social, family and emotional support of women increases, and as a result, low levels of social support can play an active role in causing depression during pregnancy. Depression in pregnant women not only threatens the health of the mother but also the health of the child. Depressive disorders may affect the child from the fetal stage; they may also affect the delivery process as well as the mother-child relationship in later years (33).

When pregnancy is associated with mental disorders, not only because of the disorder itself but also because of the social and emotional risk conditions of these women, it is a high-risk pregnancy. For this reason, the implementation of specialized services to guide the population becomes important. Primary care as an input to the health care system should be able to recognize cases in which pregnant women need follow-up by a mental health specialist, through comprehensive and qualified prenatal care.

## 5. Conclusion

Compared with low-risk women, high-risk pregnant women reported a higher prevalence of psychological symptoms. High-risk pregnant women had a significantly higher prevalence of somatization symptoms, obsessive-compulsive symptoms, depression symptoms, anxiety symptoms, hostility symptoms, and paranoid ideation than low-risk women. Therefore, educational programs during pregnancy for high-risk women can be useful.

##  Data Availability

Data supporting the findings of this study are available upon reasonable request from the corresponding author.

##  Author Contributions

M. Dalili and A. Mehdizadeh designed the study and conducted the research. M. Nodinnejad monitored, evaluated, and analyzed the results of the study. F. Karami Robati reviewed the article. All authors approved the final manuscript and take responsibility for the integrity of the data.

##  Conflict of Interest

The authors declare that there is no conflict of interest.
